# Systemic Treatment in Soft Tissue Sarcomas: Are We Making a Difference?

**DOI:** 10.3390/cancers17050889

**Published:** 2025-03-05

**Authors:** Amrit Paudel, Priya Chattopadhyay, Brandon Rose, Aleksandra Watson, Gina D’Amato, Jonathan Trent, Steven Bialick, Emily Jonczak

**Affiliations:** 1 Department of Medicine, Division of Medical Oncology, Sylvester Comprehensive Cancer Center, University of Miami, Miami, FL 33136, USA; axw859@med.miami.edu (A.W.); gina.damato@med.miami.edu (G.D.); jtrent@med.miami.edu (J.T.); steven.bialick@med.miami.edu (S.B.); 2 Department of Internal Medicine, Jackson Health System, University of Miami, Miami, FL 33136, USA; pxc804@miami.edu (P.C.); brose@med.miami.edu (B.R.)

**Keywords:** soft tissue sarcoma, chemotherapy, anthracyclines, tyrosine kinase inhibitors, immunotherapy

## Abstract

**Simple Summary:**

Sarcomas constitute a rare and heterogeneous group of malignant neoplasms, encompassing over 100 distinct histological subtypes. These tumors account for less than 1% of all adult malignancies and have propensity for diagnosis in younger individuals. Soft tissue sarcomas (STSs), originating from mesodermal tissue, can arise in a variety of structures including muscles, adipose tissue, and peripheral nerves. While anthracycline-based chemotherapy remains the cornerstone of treatment, ongoing advancements in the molecular biology of STSs are significantly enhancing our understanding of these complex tumors. These discoveries have facilitated the development of more tailored therapeutic strategies, including targeted therapies and immunotherapies, with the goal of improving treatment responses and long-term survival outcomes. However, survival rates remain suboptimal, particularly in patients with advanced-stage disease. Recent therapeutic innovations emphasize histology-specific treatment approaches, which necessitate a comprehensive integration of pathological, immunohistochemical, and molecular profiling to guide clinical decision-making. This review aims to provide an overview of the current systemic treatment options for STSs (except gastrointestinal stromal tumors), highlight recent progress in the field, and discuss emerging avenues for future therapeutic strategies in sarcoma management.

**Abstract:**

Soft tissue sarcomas [STSs] are rare tumors of mesodermal origin that arise in diverse tissues such as muscles, fat, and nerves. There are over 100 subtypes of STS, each with distinct clinical behaviors and responses to treatment. Recent advances in treatment have moved towards histology-specific approaches, emphasizing the integration of pathological, immunohistochemical, and molecular features to guide treatment. Localized STS is primarily treated with surgery, often supplemented by neoadjuvant or adjuvant radiation and/or chemotherapy. However, about half of patients with localized disease will progress to an advanced stage, which is typically managed with systemic therapies including anthracycline-based chemotherapy such as doxorubicin or epirubicin. Despite these treatments, the survival rates for most subtypes of advanced metastatic STS remain relatively low. While anthracycline-based chemotherapy remains the mainstay of treatment, ongoing research into the biology of STSs is enhancing our understanding and approach to these complex tumors with an expansion beyond chemotherapy to include targeted therapy and immunotherapy to improve response rates and survival outcomes. This review focuses on STS other than gastrointestinal stromal tumors [GISTs], examines the current systemic treatment strategies, highlights recent advances, and explores future directions in the systemic therapy of sarcoma patients.

## 1. Introduction

Soft tissue sarcomas (STSs) are rare tumors of mesodermal origin and can occur anywhere in the body. As the name implies, an STS may develop in the muscles, joints, fat, nerves, deep skin tissues, and blood vessels of the body. With more than 100 histological and molecular subtypes, an STS presents unique and diverse clinical behavior, pathology, therapy, and prognostic characteristics [1]. For example, although they are chemo-resistant, systemic therapy with KIT inhibitors such as imatinib is particularly effective in gastrointestinal stromal tumors (GISTs) which harbor KIT mutations. In contrast, leiomyosarcomas are chemo-sensitive, but typically exhibit poor responses to targeted therapies and immunotherapies [1].

Given the heterogeneity of these tumors, treatment strategies have shifted from the same-for-all approach to histology-specific approaches [2]. The release of the World Health Organization (WHO) classification for Soft Tissue and Bone Tumors in 2020 signified a major advancement in the standardization of diagnosis [3]. The update represents an agreement among a global panel of experts, including pathologists, geneticists, medical oncologists, surgeons, and radiologists. By incorporating input from expert clinicians, it underscored the crucial role of pathologic diagnosis in guiding appropriate treatment [4]. Currently, pathologic diagnosis involves the combination of morphologic, immunohistochemical, and molecular features and is a key component of clinical decision-making. The WHO classification serves as a crucial tool to foster multidisciplinary collaboration by encouraging pathologists, geneticists, and clinicians to work together to transform new pathologic discoveries into improved therapeutic strategies [4].

For localized STSs, curative intent involves surgery as the primary treatment when resection is feasible, with the addition of chemotherapy and radiation for patients at high risk for distant or local recurrence [5]. Patients with localized disease are treated with neoadjuvant chemotherapy to down-stage surgery to achieve organ preservation, to initiate earlier treatment of microscopic metastatic disease, and to understand the chemosensitivity of an individual patient’s tumor.

About half of patients with localized intermediate- or high-grade sarcomas will progress to advanced, metastatic disease [6]. For patients with locally advanced or metastatic disease, the main objective is to extend survival, primarily through systemic therapies such as cytotoxic drugs, immunotherapeutic approaches, and tyrosine kinase inhibitors [5]. The standard first-line chemotherapy for such cases includes anthracycline-based combination regimens with doxorubicin or epirubicin, or single-agent therapy due to comorbidities, which has been the primary treatment approach since the 1970s [7,8]. Unfortunately, survival outcomes continue to be suboptimal at 12.8–14.3 months for patients with advanced disease added with significant therapy-related toxicities [8]. This review focuses on the currently practiced systemic treatment of STSs other than GISTs, the overall progress in treatment, and the future direction of contemporary sarcoma therapies.

### 1.1. Epidemiology

STSs occur in approximately 1.0 to 4.0 of every 100,000 individuals worldwide [9]. Generally, the incidence of soft tissue sarcomas is higher among newborns and young children, peaking around age 5. Young adults show the lowest rates, but the incidence begins to rise steadily until around age 50, after which the rate of soft tissue sarcomas increases significantly for those over 50. According to statistics from the NCHS and SEER for the years 2004 to 2008, the average age at diagnosis for soft tissue sarcomas was 58 years. Additionally, from 2003 to 2007, the average age at death for patients with soft tissue sarcomas was 65 [10]. Overall, STSs are more prevalent in males than females [10]. According to a study of the SEER database between 1978 and 2001, the incidence rates of all types of STSs were found to be higher among black people than white people at all ages, except during childhood and among men greater than 75 years old [11]. The most common types of adult STSs are leiomyosarcomas, liposarcomas, and undifferentiated pleomorphic sarcomas [11].

### 1.2. Etiology

The majority of STSs are sporadic with no well-defined cause. This may be due to the difficulty in characterization resulting from the low statistical power of studies, which is a consequence of the disease’s low incidence and histological misclassification. Researchers have identified various associated or predisposing factors, such as genetic factors, prior radiation therapy, lymphedema, environmental carcinogens, and trauma [12]. Common heritable cancer predisposition syndromes associated with sarcomas include Familial Adenomatous Polyposis (FAP), Carney–Stratakis, Hereditary Retinoblastoma (RB1), Li–Fraumeni Syndrome, Neurofibromatosis (NF1), Werner Syndrome, Tuberous Sclerosis, Hereditary Leiomyomatosis and renal cancer (HLRCC) [13], Ollier disease, and Maffucci’s syndrome [14].

### 1.3. Prognosis

Although STSs can occur throughout the body, approximately 41% arise in the extremities, 38% in the intra-abdominal organs, 10% in the trunk, and 5% in the head or neck [12]. For patients with extremity lesions, the majority of metastases [70%] occur in the lungs [15]. Conversely, for individuals with retroperitoneal or visceral lesions, metastases are more frequently found in the liver, while the lungs serve as a secondary site [9]. Survival outcomes continue to be suboptimal at 12.8–14.3 months for patients with incurable disease [8]. The key prognostic factors for assessing the risk of local recurrence or early dissemination include tumor size, grade, and location [1]. Site-specific nomograms have been developed to predict the outcomes for extremity and retroperitoneal tumors [2,3]. According to the SEER database, the 5-year overall survival rate for soft tissue sarcomas, based on people diagnosed with soft tissue sarcomas between 2015 and 2019, was 63.4% (95% CI 61.2–65.7) for localized/regional and 12.15% (95% CI 9.4–15.6) for distant stages [16]. However, metastatic sarcomas in patients with a good performance status do have an improved overall prognosis if the tumor is sensitive to chemotherapy.

## 2. Discussion

### 2.1. Current Systemic Treatment Agents

Systemic chemotherapy remains the standard therapeutic approach for advanced-stage soft tissue sarcomas (STSs), with the primary goals being focused on symptom alleviation and the management of disease progression. Multiple systemic agents are being used in the management of STSs (Table 1). The overall response rate to cytotoxic chemotherapy in first-line treatment is approximately 25%, with a higher chemotherapy efficacy observed in younger patients, absence of liver metastasis, and patients with high-grade sarcomas [8,17].

Doxorubicin, an anthracycline which belongs to the antitumor antibiotic class of chemotherapy agents and is the first-line chemotherapy for the treatment of localized, as well as advanced or metastatic, STSs. Its use has been employed in treatment since the 1970s, with early studies as a single agent showing complete remission in 6.7% of patients and a partial remission in 20% of patients with metastatic soft tissue sarcomas [7]. Newer trials have reported a moderately improved median OS for doxorubicin monotherapy, showing 17.6 months in the GeDDiS trial [18], 16.9 months in the PICASSO III trial [19], and 19.0 months in the SARC021 trial [20].

The cumulative risk of cardiomyopathy associated with anthracyclines has long been a significant concern. However, premedication with dexrazoxane or administering doxorubicin via a 72-h infusion can reduce this risk to less than 4%, enabling the possibility of doxorubicin retreatment for most patients. Additionally, the regular monitoring of cardiac ejection fraction allows for the safe administration of doxorubicin well beyond 500 mg.

A new formulation of doxorubicin, known as liposomal doxorubicin, was developed by encapsulating the drug in liposomes modified with polyethylene glycol (PEG) [21]. Although it showed an equivalent efficacy to doxorubicin in a randomized phase II trial, both were administered at substandard doses much lower than the standard of care [22]. Doxorubicin is commonly administered in combination at 60, 75, or 90 mg/m^2^, while liposomal doxorubicin is limited to 50 mg/m^2^ due to the severe skin toxicity from the liposomes. This is an important distinction, as doxorubicin has a well-known dose–response relationship in sarcoma patients. For this reason and cost, liposomal doxorubicin is very rarely used for patients with STSs.

First synthesized in the 1960s, ifosfamide belongs to the oxazaphosphorine family of alkylating agents. Ifosfamide efficacy is 20–25% when used as monotherapy in patients with STSs [23]. A phase II trial of first-line high-dose ifosfamide in advanced soft tissue sarcomas of adults displayed a dose-dependent relationship with toxic side effects [24]. Additionally, exposure to higher doses of ifosfamide (≥10 g/m^2^ per cycle) resulted in improved complete and partial response rates, with overall response rates ranging from 19% to 37.7% [24,25]

Numerous studies have evaluated combination chemotherapy for soft tissue sarcomas, yielding varied results. A phase 3 trial by the EORTC Soft Tissue and Bone Sarcoma Group compared doxorubicin monotherapy with two combination regimens: doxorubicin plus ifosfamide (5 g/m^2^) and a four-drug regimen (cyclophosphamide, vincristine, doxorubicin, dacarbazine) [17]. This trial found no significant improvements in response rates, progression-free survival (PFS), or overall survival (OS), and exhibited increased toxicity with combination therapies [17]. Another phase 3 study compared single-agent doxorubicin with two different combination regimens, doxorubicin with ifosfamide (7.5 g/m^2^) and doxorubicin with mitomycin and cisplatin, revealing higher response rates with the combination therapy but no significant OS differences [26]. An additional study comparing doxorubicin plus dacarbazine with or without ifosfamide revealed significantly improved response rates and PFS, but not OS, with the ifosfamide-containing regimen [27]. Other studies have explored the impact of increasing dosage intensity. Trials assessing an increased dosage intensity of ifosfamide (6 g/m^2^ vs. 12 g/m^2^) alongside doxorubicin showed no benefit in either progression-free survival or overall survival [28]. Furthermore, escalating doxorubicin doses (75 mg/m^2^ vs. 50 mg/m^2^) with ifosfamide (5 g/m^2^) did not enhance response rates or OS, although it did prolong PFS [29]. A phase 3 randomized controlled trial (EORTC 62012) comparing doxorubicin alone to dose-intensified doxorubicin combined with ifosfamide as a first-line treatment for advanced or metastatic soft tissue sarcomas did not demonstrate a significant difference in overall survival [8]. However, the combination regimen (dose-intensified doxorubicin and ifosfamide) resulted in a significantly higher median progression-free survival and a better overall response [8]. Thus, dose-intensified doxorubicin with ifosfamide may be considered a viable option for palliative management in advanced soft tissue sarcomas.

Gemcitabine is a widely available pyrimidine antimetabolite with broad-spectrum efficacy against various malignancies [30]. With a generally well-tolerated toxicity profile, myelosuppression is the most frequent side effect, while non-hematological events occur relatively infrequently [30]. Docetaxel works by stabilizing tubulin, which hinders mitotic and cellular functioning. Also, docetaxel has been found to promote apoptosis and has activity in tumors that are resistant to anthracycline-derived regimens [31]. The rationale for using gemcitabine and docetaxel in combination is their potential synergy, with gemcitabine halting the cell cycle and docetaxel driving cellular apoptosis. Several clinical trials have investigated the combination of gemcitabine and docetaxel, a regimen recommended as a second-line chemotherapy treatment for STSs, and which is a well-tolerated regimen. A randomized phase II study revealed that overall survival (OS) and progression-free survival (PFS) were better with the gemcitabine–docetaxel combination compared to gemcitabine alone, with an OS of 17.9 versus 11.5 months, and a PFS of 6.2 versus 3.0 months, respectively [32]. The gemcitabine/docetaxel combination showed an improved response and survival in leiomyosarcoma, particularly in patients with a baseline WHO performance status of 0 and pleomorphic sarcomas [32,33]. A stronger tumor response was associated with improved survival. Additionally, patients with stable disease following gemcitabine/docetaxel treatment benefited from the regimen, showing an overall survival similar to that of responding patients [34].

Dacarbazine is an antineoplastic alkylating agent [35]. During the 1970s, dacarbazine emerged as one of the initial chemotherapy drugs shown to have tumor-inhibiting effects in the treatment of metastatic sarcomas [36]. Unfortunately, dacarbazine’s performance was limited due to treatment-related toxicities, with 90% of patients experiencing nausea and vomiting, and 36% developing grade 3–4 neutropenia. At that time, the lack of effective anti-emetics and leukocyte growth factors often led to the premature cessation of treatment [37]. Response rates were augmented when dacarbazine was used in combination with ifosfamide and doxorubicin [38].

In current practice, dacarbazine is frequently administered alongside anthracyclines in first-line treatment for patients with susceptible histological subtypes or when ifosfamide is not suitable. When used in advanced treatment, dacarbazine is typically given as a monotherapy [37]. A retrospective analysis from 2007 evaluated 40 patients with refractory disease treated with dacarbazine in the second or third line, showing a 20% clinical benefit rate (CBR) and a median progression-free survival (PFS) of 2 months [39]. A phase II trial conducted in 2021, using a three-drug anti-emetic regimen consisting of palonosetron or ondansetron, aprepitant, and dexamethasone, along with pegfilgrastim, demonstrated that treatment-related nausea and vomiting reduced to 37.5%, while grade 3–4 neutropenia occurred in 10% of patients, with an additional 30% clinical benefit rate [37].

Temozolomide is an imidazotetrazinone that functions as an oral prodrug, either as a standalone treatment or in combination with other cytotoxic chemotherapies, has shown efficacy in treating sarcomas [40,41]. A phase II trial assessed the efficacy of temozolomide in 25 patients with unresectable or metastatic STS, demonstrating modest results for progression-free survival [PFS] and overall survival [OS]. After a median follow-up of 13.2 months, the median PFS was 2 months, and the median OS was 13.2 months [41].

In multiple phase II trials, Trabectedin, a marine-derived drug, demonstrated efficacy in patients with metastatic STSs, and subsequently, a randomized study comparing two different doses and treatment schedules resulted in its first regulatory approval in 2007 [42,43,44,45]. A multicenter, randomized, phase III clinical trial comparing trabectedin and dacarbazine in advanced STSs demonstrated a statistically significant 45% reduction in the risk of disease progression or death with trabectedin, compared to the active control therapy, dacarbazine (*p* < 0.001) [45]. Additionally, a phase III trial comparing doxorubicin alone versus doxorubicin with trabectedin in patients with metastatic or unresectable leiomyosarcoma with no prior chemotherapy revealed longer median overall survival (Median OS, 33 months vs. 24 months) and progression-free survival (PFS, 12 months vs. 6 months) in the combination therapy group [46].

Nab-Sirolimus, an albumin-bound nanoparticle sirolimus formulation that inhibits mammalian [mechanistic] targets of rapamycin (mToR), achieved FDA approval for the treatment of malignant perivascular epithelioid cell tumors (PEComa) based on positive results from the phase II, single-arm, registration trial AMPECT, NCT02494570 [47]. The table below (Table 2) provides a comprehensive overview of the above-mentioned chemotherapy trials.

### 2.2. Neoadjuvant Chemotherapy

Several prospective and retrospective studies have demonstrated the benefits of incorporating chemotherapy into the neoadjuvant treatment of high-grade soft tissue sarcomas (STS), including significant reductions in distant metastases and improvements in disease-free and overall survival [48,49,50,51,52,53].

A combined analysis of the Radiation Therapy Oncology Group (RTOG-9514) study (NCT00002791), which investigated neoadjuvant chemoradiation, and the RTOG-0630 study (NCT00589121), which evaluated neoadjuvant radiation therapy alone, revealed pathological complete response rates (defined as a 0% tumor viability in the final specimen after neoadjuvant treatment) of 27.5% for neoadjuvant chemoradiation (RTOG-9514) and 19.4% for neoadjuvant radiation therapy (RTOG-0630) alone [53]. In RTOG 9514, patients received 6 cycles of mesna, doxorubicin, ifosfamide, and dacarbazine chemotherapy interdigitated with 44 Gy RT. The overall survival (OS) rate was 100% for patients, achieving a pathological complete response at a median follow-up of over 5 years. With no pathological complete response, the 5-year survival rates were 76.5% (95% CI, 62.3–90.8%) in the RTOG-9514 study and 56.4% (95% CI, 43.3–69.5%) in the RTOG-0630 study [53]. In terms of histotype-tailored chemotherapy, a phase III ISG-STS1001 study (NCT01710176) compared a neoadjuvant histotype-tailored chemotherapy regimen over the standard chemotherapy [52]. The standard chemotherapy consisted of epirubicin plus ifosfamide. Histotype-tailored chemotherapy consisted of trebectedin for high-grade myxoid liposarcoma, gemcitabine plus dacarbazine for leiomyosarcoma, high-dose ifosfamide for synovial sarcoma, etoposide plus ifosfamide for malignant peripheral nerve sheath tumors, and gemcitabine plus docetaxel for undifferentiated pleomorphic sarcomas. The study did not show any benefit from neoadjuvant histotype-tailored therapy over standard chemotherapy in high-risk soft tissue sarcomas [52].

### 2.3. Adjuvant Chemotherapy

Several research groups have investigated the efficacy of adjuvant chemotherapy following primary surgery for patients with high- or high/intermediate-grade soft tissue sarcomas (STSs). However, numerous prospective randomized trials have not definitively established the benefit of adjuvant chemotherapy in adults with resectable STSs [54,55,56,57,58,59,60,61,62].

In a study by the European Organisation for Research and Treatment of Cancer (EORTC), the researchers analyzed individual patient data from the EORTC-62931 and EORTC-62771 trials. The chemotherapy regimen for EORTC-62931 included doxorubicin and ifosfamide combined with lenograstim [63], while the EORTC-62771 trial consisted of doxorubicin, dacarbazine, cyclophosphamide, and vincristine (CYVADIC) [62,64]. Adjuvant chemotherapy did not show a significant impact on OS, but was associated with improved relapse-free survival (RFS) [64]. Histological grade, tumor size, and quality of resection were recognized as independent prognostic factors for relapse-free survival (RFS) and overall survival (OS) [64].

One of the major challenges in assessing the adjuvant treatment efficacy for STSs is the considerable heterogeneity among patients, including the variability in histological subtypes, tumor sites, tumor size, and histological grades. Additionally, many of the trials are limited by their relatively small sample sizes and substantial interstudy variability.

### 2.4. Chemotherapy for Advanced Disease

Clinical practice for managing patients with locally advanced and metastatic STSs exhibits considerable variation. First-line systemic therapy for most cases of locally advanced and metastatic STSs primarily consists of an anthracycline-based regimen. The benefit of adding additional drugs to a single-agent doxorubicin regimen remains a topic of ongoing debate.

A randomized, three-armed, phase III study compared doxorubicin alone, ifosfamide with doxorubicin, and the third arm was mitomycin, doxorubicin, and cisplatin. The combination of ifosfamide and doxorubicin demonstrated a significantly higher response rate compared to doxorubicin alone. The combination of mitomycin, doxorubicin, and cisplatin also showed greater activity than single-agent doxorubicin. However, these combinations were associated with more severe myelosuppression compared to doxorubicin alone or the three-drug regimen [26].

Similarly, a phase III randomized study evaluated doxorubicin and dacarbazine with or without ifosfamide. The addition of ifosfamide resulted in a higher response rate (17% vs. 32%; *p* < 0.002) and a longer time to progression (4 months vs. 6 months; *p* < 0.02). However, the overall survival advantage for the two-drug regimen compared to the combination with ifosfamide was not statistically significant in a multivariate analysis (12 months vs. 13 months; *p* = 0.04) [27].

For second line and subsequent treatments for soft tissue sarcomas, several chemotherapy agents have been studied and utilized, as described in detail above. Liposomal doxorubicin, which exhibits a similar efficacy to doxorubicin, offers a more favorable toxicity profile [22]. Gemcitabine combined with docetaxel is another alternative that has shown some benefit [33]. Trabectedin is specifically utilized for advanced liposarcomas and leiomyosarcomas [45]. Additionally, eribulin, a microtubule inhibitor, is used in leiomyosarcomas and received FDA approval in 2016 for the treatment of unresectable or metastatic liposarcomas [65].

### 2.5. Targeted Agents

Outside the realm of GISTs, tyrosine kinase inhibitors (TKIs) have been successfully utilized in management of other STSs such as DFSP (with no fibrosarcomatous differentiation) and desmoid tumors. In 2012, the FDA approved Pazopanib, an oral TKI for advanced, previously treated, non-adipocyte STSs, based on the results from the PALETTE phase III placebo-controlled trial [66]. Sorafenib is an oral multitargeted TKI that primarily inhibits VEGFR and PDGFR. The results from phase II clinical trials indicated that Sorafenib had moderate activity as a second-line therapy for metastatic STSs [67,68]. Additionally, in a double-blind phase III trial, Sorafenib significantly extended the progression-free survival for patients with desmoid tumors [69].

The DeFi (Desmoid Fibromatosis) trial studied Nirogacestat in adults with progressing desmoid tumors. Nirogacestat, an oral gamma secretase inhibitor, had a significant progression-free survival benefit over a placebo [70]. TKIs like Sorafenib, Sunitinib, and Pazopanib have also been utilized with success in initial treatment or after progression on Nirogacestat [69,71,72]. Tazametostat, a potent and selective inhibitor of histone methyltransferase EZH2 (enhancer of zeste homolog 2), with some activity against EZH2 gain-of-function mutations (including Y646X and A687V, as well as EZH1), received FDA approval for use in advanced epithelioid sarcomas characterized by the loss of INI1/SMARCB1 based on positive results from the open-label, multicenter trial (Study EZH-202, NCT02601950) [73]. The study of neurotrophic tyrosine receptor kinase (NTRK) fusions in sarcomas has become increasingly significant due to the therapeutic implications of targeting these genetic abnormalities with TRK inhibitors. A subset analysis of three clinical trials, NCT02122913, NCT02637687 (SCOUT), and NCT02576431 (NAVIGATE), revealed that over half of patients had a durable response (an objective response rate of 58% (95% confidence interval, 41–74)) to larotrectinib, with no unexpected side effects [74]. Anaplastic lymphoma kinase (ALK) gene rearrangement is present in approximately 50% of inflammatory myofibroblastic tumors (IMTs), which are rare mesenchymal tumors primarily affecting pediatric and adolescent populations. In July 2022, the FDA approved Crizotinib, a tyrosine kinase receptor inhibitor that targets ALK, for use in adult and pediatric patients aged one year and older with unresectable, recurrent, or refractory ALK-positive IMTs. This approval was based on the findings from two multicenter, single-arm, open-label trials: ADVL0912 (NCT00939770) and A8081013 (NCT01121588) [75,76].

### 2.6. Immunotherapy

Immunotherapy in the management of STSs continues to be under investigation. Approximately 50% of sarcomas, notably leiomyosarcomas, chondrosarcomas, liposarcomas, and UPS, expressed programmed death ligand-1 (PD-L1) based on immunohistochemistry and presented PD-1+ Tumor-Infiltrating Lymphocytes (TIL) [77]. The bio-clinical relevance of PD-1 and PD-L1 remains an area that needs further investigation in sarcomas, mainly due to their high heterogeneity.

Large series of STSs revealed that most STS subtypes show the expression of both PD-1 and PDl-1 factors [78,79]. In the STS cohort of the SARC028 trial, an objective response was noted with Pembrolizumab (anti-PD-1 antibody) in undifferentiated pleomorphic sarcomas (UPS), liposarcomas, and synovial sarcomas [78]. No patients with leiomyosarcomas had an objective response in the same study [80]. Additionally, the SARC028 expansion cohort analysis confirmed the clinical activity of Pembrolizumab in undifferentiated pleomorphic sarcomas (UPS) and dedifferentiated liposarcomas (DDLPS) [81].

Atezolizumab, an anti-programmed death ligand-1 (PD-L1) agent, received FDA approval for the treatment of adult and pediatric patients aged 2 years and older with unresectable or metastatic alveolar soft-part sarcomas (ASPS), following promising results from an investigator-initiated, multicenter, single-group, phase 2 study [82]. Additionally, the AcSé Pembrolizumab study, a phase 2 basket trial assessing the efficacy of Pembrolizumab monotherapy in rare cancers, revealed varied responses across different sarcoma subtypes. Specifically, ASPS showed a partial response rate of 50%, while SMARCA4-deficient sarcomas and malignant rhabdoid tumors exhibited a 25% partial response rate [83]. Notably, two complete responses were observed in patients with ASPS and epithelioid sarcomas [83].

Nivolumab (anti-PD-1 antibody) monotherapy and in combination with ipilimumab (anti-CTLA-4 antibody) was evaluated by the phase II Alliance trial in locally advanced, unresectable, or metastatic sarcomas. With combination therapy, the ORR was 16%, and responses were seen in LMS, myxofibrosarcomas (MFS), UPS, and Angiosarcomas [84]. The efficacy of ipilimumab and nivolumab in Angiosarcomas was also demonstrated in the multicenter, phase II DART trial (ORR 25%) [85]. Combinatorial strategies [chemotherapy, radiotherapy, tyrosine kinase inhibitors] and novel agents are in development with several ongoing studies.

## 3. Future Directions

Current multimodal treatment concepts for soft tissue sarcomas combine surgery, polychemotherapy (with/without local hyperthermia), irradiation, immunotherapy, and/or targeted therapeutics. Conventional chemotherapy remains fundamental in the treatment of many soft tissue sarcomas. Similar to other solid tumors, advancements in targeted therapy and immunotherapy underscore the growing significance of molecular testing in guiding precise diagnostic strategies and targeted therapies within the STS domain. Common genetic alterations and genes affected in STSs have been described in the literature (Table 3).

In well-differentiated and dedifferentiated liposarcomas, MDM2 amplification is frequently observed [86]. Various clinical trials are currently investigating therapeutic strategies aimed at inhibiting MDM2, including small-molecule inhibitors, antisense oligonucleotides, and peptides designed to disrupt the MDM2–p53 interaction, highlighting its potential as a viable approach for soft tissue sarcoma treatment [99].

Cyclin-dependent kinase 4 (CDK4) amplification occurs in over 90% of well-differentiated and dedifferentiated liposarcomas. Preclinical studies and a phase II clinical trial of palbociclib have demonstrated its antitumor activity, resulting in improved progression-free survival in patients with advanced or metastatic well-differentiated and dedifferentiated liposarcomas [100].

Other notable targets being investigated in the STS realm include NTRK fusion in CD34-positive fibrosarcomas of bone and soft tissue [101,102,103], EZH2/INI1 loss (epithelioid sarcomas, rhabdoid tumors) [104], and ALK fusion in inflammatory myofibroblastic tumors [105].

Chimeric antigen receptor [CAR] T cell therapy, an innovative therapeutic approach, involves the genetic engineering of T cells to express a chimeric antigen receptor, which is constructed by linking a single-chain variable fragment [scFv] derived from a specific antibody to the T cell receptor. This engineered receptor is designed to bind to a particular tumor-associated antigen (TAA) present on cancer cells [106]. CAR-T has demonstrated significant efficacy in the treatment of B-cell lymphoma and acute lymphoblastic leukemia. Recent advancements have identified several sarcoma-associated antigens (SAAs) that are potentially targetable by CAR T cell therapy, yielding promising outcomes in preclinical and clinical studies [107]. Notable SAAs under investigation in STSs with encouraging results include human epidermal growth factor receptor 2 (HER2) [108,109], disialoganglioside (GD2) [110,111], receptor tyrosine kinase-like orphan receptor 1 (ROR1) [112], and CD44v6 [113].

Afamitresgene autoleucel treatment demonstrated durable responses in heavily pre-treated patients with HLA-A*02 and MAGE-A4-expressing synovial sarcoma. The open-label, non-randomized Phase 2 trial (SPEARHEAD) involved patients with synovial sarcoma and myxoid round-cell liposarcoma. In the study, the overall response rate (ORR) was 37% (19 of 52 patients; 95% CI 24–51). Specifically, the ORR was 39% (17 of 44 patients; 95% CI 24–55), median duration of response was 11.6 months (95% CI 4.4–18.0) in patients with synovial sarcoma, leading to the accelerated FDA approval. In patients with myxoid round cell liposarcoma, ORR of 25% (2 of 8 patients; 95% CI 3–65), median duration of response 4.2 months (2.9–5.5) [114]. Immune-based approach including replication-competent oncolytic virus could potentially be another alternative tool that continues to be an area of ongoing research. Various ongoing trials are investigating the utility of oncolytic viruses in the management of sarcoma. Notable virus backbones with agents being investigated in sarcoma include Herpes Simplex Virus-1 (talimogene laherparepvec, T-VEC) [115], Vaccinia Virus (intratumoral Pexa-Vec JX-594) [116] and Seneca Valley Virus (NTX-010) [117].

## 4. Conclusions

Sarcomas are a rare yet highly diverse group of malignant neoplasms, encompassing over 100 distinct histological subtypes and accounting for less than 1% of adult malignancies. Soft tissue sarcomas (STSs), a major subset, display unique clinical behaviors, histopathological features, and prognostic factors. Despite this considerable heterogeneity, anthracycline-based chemotherapy remains the cornerstone of treatment for most metastatic STSs, as well as in both adjuvant and neoadjuvant settings, often in combination with other chemotherapeutic agents. Recent advances in genetic sequencing have highlighted the significant genetic diversity within tumors of the same histological subtype, complicating the classification and treatment of sarcomas. These advancements in genetic profiling and cytogenetics have broadened our understanding of the complexity and heterogeneity of these tumors, resulting in an increased focus on developing targeted therapeutic approaches that are based on the molecular and genetic profiles of individual tumors.

## Figures and Tables

**Table 1:** Current systemic treatment modalities.

Treatment Modalities	Agents
Chemotherapy	Doxorubicin Ifosfamide Gemcitabine Docetaxel Dacarbazine Temozolomide Trabectedin Eribulin Vincristine Cyclophosphamide Irinotecan Topotecan Epirubicin Etoposide Vinorelbine Liposomal doxorubicin Paclitaxel Methotrexate
Targeted Therapy	Multitargeted Tyrosine Kinase Inhibitors Imatinib Sunitinib Sorafenib Regorafenib Ripretinib Avapritinib Nilotinib Pazopanib Cabozantinib Axitinib Lenvatinib Immunotherapy Nivolumab Ipilimumab Pembrolizumab Atezolizumab CDK4/6 Inhibitors Palbociclib Ribociclib Abemaciclib mTOR Inhibitors Sirolimus Temsirolimus Everolimus Nab-Sirolimus VEGF Inhibitors Bevacizumab PARP Inhibitors Olaparib Rucaparib Niraparib Talazoparib EZH-1 Inhibitors Tazemetostat Gamma-Secretase Inhibitors Nirogacestat NTRK Inhibitors Larotrectinib Entrectinib Repotrectinib ALK Inhibitors Lorlatinib Crizotinib Alectinib Brigatinib Ceritinib CSF1R Inhibitor Pexidartinib
Hormonal Therapy	Aromatase Inhibitors Tamoxifen
T Cell Therapy	Afamitresgene autoleucel

CDK: cyclin-dependent kinase, mTOR: mechanistic target of rapamycin, VEGF: Vascular Endothelial Growth Factor, PARP: Poly [ADP-Ribose] Polymerase, EZH: enhancer of zeste homolog, NTRK: neurotrophic tyrosine receptor kinase, ALK: anaplastic lymphoma kinase, CSF1R: Colony Stimulating Factor 1 Receptor.

**Table 2:** Comprehensive Overview of Chemotherapy Trials in STS.

Trial	Year	Study Design	Population	Intervention	Findings	Adverse Events
EORTC 62012 [8]	2014	Phase III RCT	-Advanced/metastatic STS -All Sarcoma Subtypes	Doxorubicin (75 mg/m^2^) vs. Doxorubicin (75 mg/m^2^) + Ifosfamide (10 g/m²)	Median OS: 12.8 vs. 14.3 months Median PFS: 4.6 vs. 7.4 months; ORR: 14% vs. 26%	Grade 3-4: Leucopenia (18% vs. 43%), Neutropenia (37% vs. 42%), Febrile Neutropenia (13% vs. 46%), Anemia (5% vs. 35%), Thrombocytopenia (1% vs. 33%)
GeDDiS [18]	2017	Phase III RCT	-Advanced/metastatic STS -Various STS subtypes	Doxorubicin (75 mg/m^2^) vs. Gemcitabine (675 mg/m² D1, D8) + Docetaxel (75 mg/m^2^)	Median PFS: 23.3 vs. 23.7 weeks Median OS: 76.3 vs. 67.3 weeks	Grade 3-4: Neutropenia (25% vs. 20%), Febrile Neutropenia (21% vs. 15%), Fatigue (6% vs. 14%), Oral Mucositis (14% vs. 2%), Pain (8% vs. 10%)
PICASSO III [19]	2016	Phase III RCT	-Metastatic STS -Various STS subtypes	Doxorubicin (75 mg/m^2^) vs. Doxorubicin (75 mg/m^2^) + Palifosfamide (150 mg/m^2^, D1–D3)	Median OS: 16.9 vs. 15.9 months Median PFS: 5.2 vs. 6.0 months	Grade 3-4: Febrile Neutropenia (21.4% vs. 12.6%)
SARC021 [20]	2017	Phase III RCT	-Advanced/metastatic STS -Various STS subtypes	Doxorubicin (75 mg/m^2^) vs. Doxorubicin (75 mg/m^2^) + Evofosfamide (300 mg/m^2^ D1, D8)	Median OS: 19 vs. 18.4 months	Grade 3-4: Anemia (21% vs. 48%), Neutropenia (29% vs. 15%), Febrile Neutropenia (11% vs. 18%), Thrombocytopenia (1% vs. 14%),
Maki et al. [32]	2007	Phase II RCT	-Metastatic STS -Various STS subtypes	Gemcitabine (1200 mg/m^2^ D1, D8) Vs Gemcitabine (900 mg/m^2^ D1, D8)+ Docetaxel (100 mg/m^2^ D8)	Median PFS: 3 months vs. 6.2 months Median OS: 11.5 months vs. 17.9 months	Grade 3-4: Neutropenia (28% vs. 16%), Febrile Neutropenia (7% vs. 5%), Grade 3 Anemia (13% vs. 7%), Thrombocytopenia (35% vs. 40%),
Demetri et al. [45]	2016	Phase III RCT	-Metastatic Liposarcoma and Leiomyosarcoma	Trabectedin (1.5 mg/m^2^) vs. Dacarbazine (1 g/m^2^)	Median PFS: 4.2 vs. 1.5 months Median OS: 12.4 vs. 12.9 months	Grade 3-4: Neutropenia (35% vs. 21%), Anemia (14% vs. 12%), Elevated ALT (26% vs. 1%)
Talbot et al. [41]	2003	Phase II	Unresectable/Recurrent/Metastatic STS -Various STS subtypes	Temozolomide (200 mg/m^2^ q12h for 5 days followed by 9 doses 90 mg/m^2^ q4weeks)	Objective Response Rate: 8% Median OS: 13.2 months For LMS subgroup: Median PFS: 3.9 months, Median OS: 30.8 months	Grade 3: Nausea (4%), Anemia (4%)
AMPECT [47]	2021	Phase II	Malignant PEComa (metastatic or locally advanced, ineligible for surgery)	Nab-Sirolimus (100 mg/m^2^ D1, D8)	Objective Response Rate: 39% Median PFS: 10.6 months Median OS: 40.8 months	Grade 1-2: Anemia 47%, Thrombocytopenia 32%, Mucositis 79%, Fatigue 59%, Rash 56%
LMS04 [46]	2024	Phase III RCT	Leiomyosarcoma	Doxorubicin (75 mg/m^2^) vs. Doxorubicin (75 mg/m^2^) + Trabectedin (1.5 mg/m^2^)	Median OS: 33 vs. 24 months Median PFS: 12 vs. 6 months	Grade 3-4: Neutropenia (13% vs. 80%), Anemia (4% vs. 31%), Febrile neutropenia (9% vs. 28%)

RCT: Randomized Controlled Trial, D: Day.

**Table 3:** Genetic alteration, STS subtypes, and genes affected.

Genetic Alterations	STS Subtype with Genes Affected
Amplification	Liposarcoma-MDM2 [86,87], CDK4 [86,87], HMGA2 [87], c-JUN [87] Fibrosarcoma-MDM2 [88] Leiomyosarcoma-MYOCD [89]
Deletion	Leiomyosarcoma-PTEN [90,91], RB1 [91] Embryonal Rhabdomyosarcoma-CDKN2A/B [92] Undifferentiated pleomorphic sarcoma-RB1 [93,94]
Mutations	Leiomyosarcoma-TP53 [91,95,96], ATRX [91,95,96], MED12 [96] Angiosarcoma-TP53 [97], PTRB [97] MPNST-NF1 [98], CDKN2A [98], TP53 [98]
Overexpression	Angiosarcoma-VEGF [97]

MDM2: murine double minute 2, CDK4: cyclin-dependent kinase 4, HMGA2: High Mobility Group AT-Hook 2, c-JUN: Jun Proto-Oncogene, MYOCD: myocardin, PTEN: Phosphatase and Tensin Homolog, RB1: Retinoblastoma 1, CDKN2A/B: cyclin-dependent kinase inhibitor 2A/B, TP53: Tumor Protein 53, NF1: Neurofibromin, VEGF: Vascular Endothelial Growth Factor.
